# Facile Designed Manganese Oxide/Biochar for Efficient Salinity Gradient Energy Recovery in Concentration Flow Cells and Influences of Mono/Multivalent Ions

**DOI:** 10.1021/acsami.0c21956

**Published:** 2021-04-23

**Authors:** Guangcai Tan, Nan Xu, Dingxue Gao, Xiuping Zhu

**Affiliations:** ‡ Department of Civil and Environmental Engineering, Louisiana State University, Baton Rouge, Louisiana 70803, United States; † CAS Key Laboratory of Urban Pollutant Conversion, Department of Environmental Science and Engineering, University of Science and Technology of China, Hefei 230026, China; § Shenzhen Engineering Research Center for Nanoporous Water Treatment Materials, School of Environment and Energy, Peking University Shenzhen Graduate School, Shenzhen 518055, China

**Keywords:** biochar, manganese oxide, salinity gradient energy, concentration flow cell, mono/multivalent ions

## Abstract

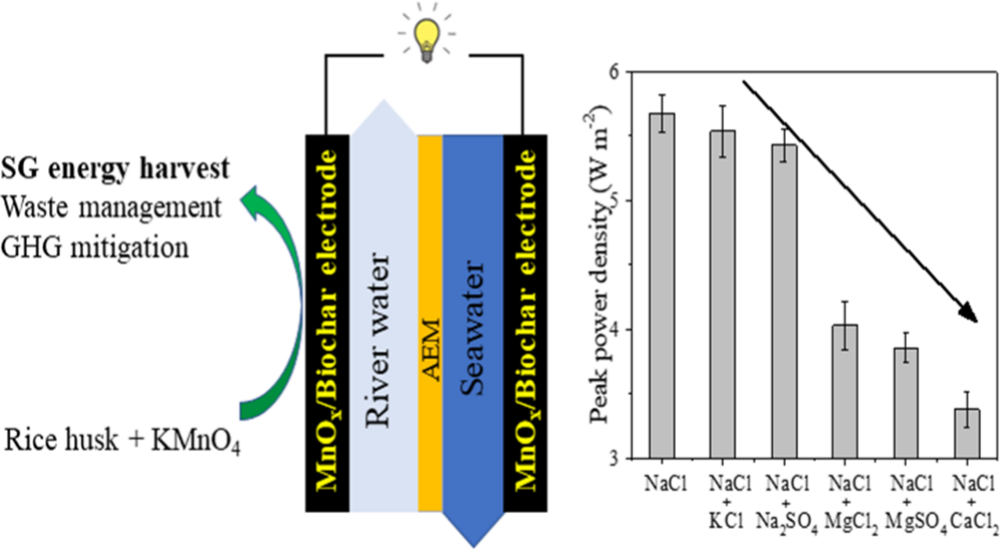

Development of effective, environmentally friendly, facile large-scale processing, and low-cost materials is critical for renewable energy production. Here, MnO*_x_*/biochar composites were synthesized by a simple pyrolysis method and showed high performance for salinity gradient (SG) energy harvest in concentration flow cells (CFCs). The peak power density of CFCs with MnO*_x_*/biochar electrodes was up to 5.67 W m^–2^ (ave. = 0.91 W m^–2^) and stabilized for 500 cycles when using 1 and 30 g L^–1^ NaCl, which was attributed to their high specific capacitances and low electrode resistances. This power output was higher than all other reported MnO_2_ electrodes for SG energy harvest due to the synergistic effects between MnO*_x_* and biochar. When using a mixture with a molar fraction of 90% NaCl and 10% KCl (or Na_2_SO_4_, MgCl_2_, MgSO_4_, and CaCl_2_) in both feed solutions, the peak power density decreased by 2.3–40.1% compared to 100% NaCl solution with Ca^2+^ and Mg^2+^ showing the most pronounced negative effects. Our results demonstrated that the facile designed MnO*_x_*/biochar composite can be used for efficient SG energy recovery in CFCs with good stability, low cost, and less environmental impacts. When using natural waters as the feed solutions, pretreatment would be needed.

## 1 Introduction

Global warming is a critical issue for the sustainable development of the world. To avoid the emission of greenhouse gases from combustion of fossil fuels, many kinds of renewable resources have been extensively studied, such as solar, geothermal, and wind energy. Salinity gradient (SG) energy (also called osmotic power) is a less well-known renewable resource, which is released when two solutions with different salt concentrations are mixed due to different chemical potentials.^1,2^ SG energy can be harvested from natural origins (e.g., in estuaries where river water flows into the ocean driven by the global hydrological cycle)^3^ or anthropogenic streams (e.g., by combining the low salinity effluent from wastewater treatment and the rejected brine from desalination plants).^4^ It is estimated that the power production from SG energy can be around 1.4–2.6 TW globally.^3^ Additionally, SG energy is much more predictable and continuously available compared to intermittent renewable energy (e.g., tidal, solar, and wind).

Several technologies have been developed to harvest SG energy, such as pressure-retarded osmosis (PRO),^5,6^ reverse electrodialysis (RED),^7^ capacitive mixing (CapMix),^8,9^ and hydrogel expansion (HEx).^10^ Another new emerging method for SG energy harvest was the employment of concentration flow cells (CFCs), which combined the mechanisms responsive for power production of RED (Donnan Potential) and CapMix (electrode potential), and consequently showed a high power density of up to 12.6 W m^–2^ membrane.^11^ The first CFC was constructed with two identical electrodes (copper hexacyanoferrate, CuHCF), which were separated by an anion-exchange membrane (AEM).^11^ One side was fed with synthetic freshwater (1 g L^–1^ NaCl) and another side was fed with synthetic seawater (30 g L^–1^ NaCl). The solution paths could be periodically switched to recharge the CFCs to continuously generate power.^11^ Later, bismuth chloride (BiCl_3_),^12^ bismuth oxychloride (BiOCl),^13^ molybdenum disulfide (MoS_2_),^14^ and carbonized peat moss^15^ were also employed for efficient SG energy harvest in CFCs. However, the preparations of CuHCF, BiCl_3_, BiOCl, and MoS_2_ were relatively complex with high costs. They are also not so stable due to the volume expansion that resulted from Na^+^ or Cl^–^ insertions, which would release toxic metals (e.g., Cu^2+^, Bi^3+^, and Mo^2+^) into the environment. The synthesis of carbonized peat moss was relatively simple but needed to be treated with HCl and NaOH, generating large amounts of acid–alkali wastes. Moreover, most studies on SG energy recovery used synthetic river water and seawater, which only contain NaCl,^11,16^ but the natural seawater includes other inorganic solutes (e.g., K^+^, Mg^2+^, Ca^2+^, and SO_4_^2–^) with more than 10% weight.^17,18^ It is important to figure out the possible negative impacts of inorganic ions on power output for SG energy harvest.

Biochar is produced from pyrolysis of waste biomass (e.g., forest waste, straw, rice husk, and animal waste) under oxygen-limited conditions.^19,20^ Attributed to its rich carbon contents, abundant sources, various functional groups (e.g., carbonyl, hydroxyl, and carboxyl), and chemical stability, biochar has been proved to be an efficient material for waste management, greenhouse gas mitigation, soil amendment, and environmental remediation.^20−22^ Moreover, biochar can be used as a sustainable carbon platform that combines with nanosized metal oxides (i.e., NiO*_x_*, MgO, MnO*_x_*, and Fe_3_O_4_/γ-Fe_2_O_3_) during pyrolysis processes to obtain the metal oxide/biochar composites.^19,22,23^ Compared to unmodified biochar, the facile synthesized metal oxide/biochar composites usually have higher surface areas, better pore features, new structures, and enhanced specific capacitances, which have been already successfully used for energy generation, storage, and conversion.^19,22^ For example, the Ni-loaded biochar was proved to be an efficient and very stable supercapacitor.^24^ The Mn (or Fe)-loaded biochar was demonstrated to be sustainable alternatives in Li-ion batteries as anode materials.^25,26^ Recently, Fortunato *et al.*(27) reported 12 different types of MnO_2_ as electrode materials for SG energy harvest in CFCs. A maximum peak power density of 4 W m^–2^ and an average power density of 0.8 W m^–2^ were obtained with δ-MnO_2_. The low conductivity (<10^–5^ S cm^–1^) and poor long-term charge/discharge stability of MnO_2_ limit its performance for SG energy harvest.^28^

Here, MnO*_x_*/biochar composites were synthesized by pyrolyzing dried rice husk soaked with KMnO_4_ in one step, which is very simple and easy for large-scale processing with low costs. Moreover, the CFCs with MnO*_x_*/biochar electrodes had high power output and good stability. The maximum power density was 5.67 W m^–2^ using 1 and 30 g L^–1^ NaCl over 500 cycles, which was higher than CFCs with other MnO*_x_* electrodes (4 W m^–2^) and CapMix (0.2 W m^–2^) (Table S1). In the presence of other inorganic ions (K^+^, Mg^2+^, Ca^2+^, and SO_4_^2–^), both the open-circuit voltage and power output were inhibited compared to the case when only NaCl was in feed solutions. It indicated that pretreatment would be needed to obtain better power output when using natural feed waters.

## 2 Materials and Methods

### 2.1 Electrode Preparation

To synthesize MnO*_x_*/biochar composites, 10 g of dried rice husk (*Oryza sativa* L.) was soaked in 100 mL of 0.5 M KMnO_4_ solution overnight, sonicated for 2 h, and dried at 70 °C for 1 day. The KMnO_4_-treated rice husk was then subjected to pyrolysis in a tube furnace (GSL-1500X, MTI, USA) under a N_2_-protected atmosphere at different peak temperatures (400, 500, 600, and 700 °C) for 1.5 h. The samples were finally rinsed with deionized (DI) water, filtrated, and dried overnight at 50 °C under vacuum. The obtained MnO*_x_*/biochar composites were designated as BioMn400, BioMn500, BioMn600, and BioMn700. For comparison, unmodified rice husk biochar was made in the same way but without presoaking of the rice husks in KMnO_4_ solution, which are designated as Bio400, Bio500, Bio600, and Bio700. The procedure for electrode preparation was reported in our previous studies,^12,13^ and the physiochemical and electrochemical characterization methods of prepared electrodes are shown in the Supporting Information.

### 2.2 Construction and Performance Tests of Concentration Flow Cells

The configuration and performance tests of CFCs were similar to our previous studies (Figure S1).^12,13^ The two identical water chambers of CFCs were 0.67 cm × 3 cm × 127 μm. To test the performance of CFCs with different MnO*_x_*/biochar electrodes, high-concentration (HC, 30 g L^–1^ or 0.513 M) and low-concentration (LC, 1 g L^–1^ or 0.017 mM) NaCl solutions were used.

The HC and LC solutions were alternatingly pumped through the two channels, and the flow rate was 15 mL min^–1^. The flow orientation of LC and HC solutions along the membrane was counterflow (Figure S1). To test the power output, different external resistors (*R*_ext_ = 5–50 Ω) were connected between the two electrodes. The 3-way switching valves were used to switch the solutions. Cell voltage (*U*, in V) and open-circuit voltages (OCVs, in V) of the CFCs were measured through a potentiostat (Bio-Logic, France). Instantaneous power density (*P*_ins_, in W m^–2^) was obtained based on cell voltage, external resistance, and the area of the working membrane (*A* ≈ 2 cm^2^): *P*_ins_ = *U*^2^/*R*_ext_/*A*. The average power density (*P*_ave_, in W m^–2^) and energy density (*E*_d_, in J m^–2^) were calculated based on the instantaneous’ power densities over one cycle (*P*_ave._ = ∫_0_^*t*_cycle_^*P*_ins._d*t*/*t*_cycle_ and *E*_d_ = ∫_0_^*t*_cycle_^*P*_ins._d*t*, where *t*_cycle_ is the cycle time).

To test the feasibility for SG energy harvest from brines, different combinations of HC (30, 100, 200, or 300 g L^–1^) and fixed LC (1.0 g L^–1^) NaCl solutions were employed for the CFC with BioMn600 electrodes for power generation. To test the stability, the CFC with BioMn600 electrodes was run at 6 Ω (optimum external resistor) for 500 cycles. Both the power and the pH of the outflow were recorded over the whole 500 cycles. To investigate the effects of inorganic ions, 10% (in molar) of NaCl was replaced with KCl, Na_2_SO_4_, MgCl_2_, MgSO_4_, or CaCl_2_·2H_2_O (VWR, USA) for both HC and LC solutions fed into the CFC with BioMn600 electrodes. Therefore, the molarity of the feed solutions was maintained as a constant, and the conductivity was consistent to a certain degree. For example, when investigating the mixture of MgSO_4_ with NaCl, 10% of the dissolved salts was MgSO_4_ (0.0513 M in HC and 0.0017 M in LC), and the remaining 90% was NaCl (0.4617 M in HC and 0.0153 M in LC). Therefore, the total molar salt concentration was always 0.513 M for HC and 0.017 M for LC. Table S2 gives the physicochemical properties of the studied ions.

## 3 Results and Discussion

### 3.1 Physiochemical and Electrochemical Characterizations of MnO*_x_*/Biochar

**Figure 1:**
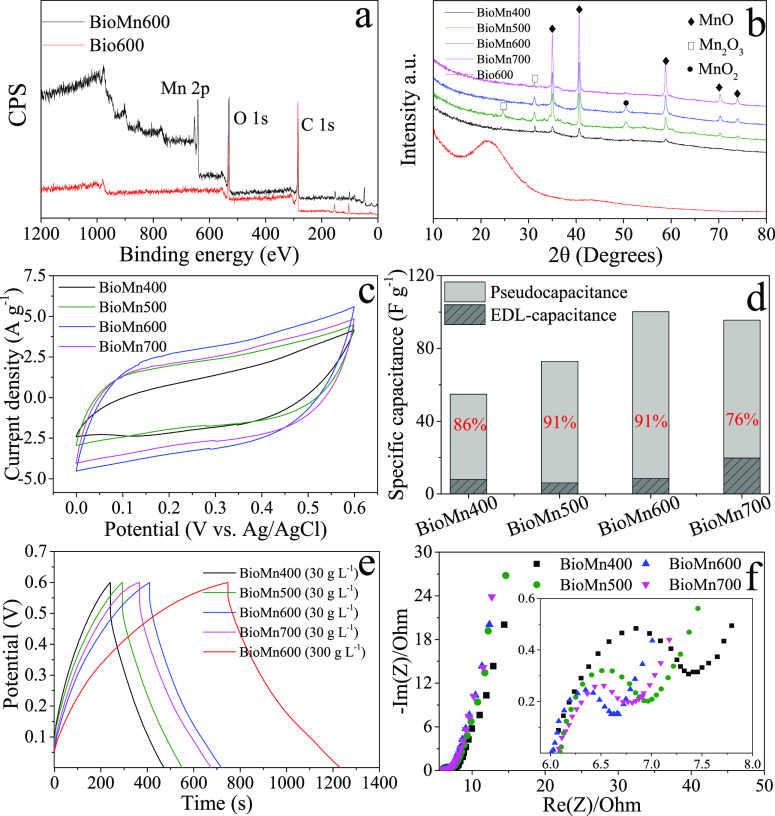
(a) XPS survey spectra of Bio600 and BioMn600, (b) X-ray diffraction patterns of MnO*_x_*/biochar and Bio600, (c) cyclic voltammograms of MnO*_x_*/biochar electrodes at a scan rate of 5 mV s^–1^, (d) pseudocapacitance and EDL capacitance contributions to specific capacitances of MnO*_x_*/biochar electrodes at the potential of 0.6 V and the scan rate of 5 mV s^–1^, (e) representative galvanostatic charge/discharge profiles of MnO*_x_*/biochar electrodes in 30 g L^–1^ NaCl solutions and the BioMn600 electrode in 300 g L^–1^ NaCl solutions at a current density of 0.2 A g^–1^, and (f) Nyquist plots for impedance responses of MnO*_x_*/biochar electrodes (inset shows a close-up of the near origin section).

The surface area and porosities of MnO*_x_*/biochar composites are given in Table 1. As the pyrolysis temperatures increased from 400 to 600 °C, the BET surface areas increased from 81.58 (BioMn400) to 280.8 m^2^ g^–1^ (BioMn600), while they slightly decreased to 263.2 m^2^ g^–1^ for BioMn700. Correspondingly, BioMn600 possessed the largest average pore diameter and total pore volume. The strong peak of Mn that showed in the XPS (Figure 1a) and SEM–EDS (Figure S2) spectra and the Mn–O bond that showed near 511 cm^–1^ in the FTIR spectra of all MnO*_x_*/biochar composites (Figure S3) proved the existence of Mn on the surface of MnO*_x_*/biochar composites, which was consistent with previous studies that made MnO*_x_* particles from KMnO_4_ under high temperatures.^29,30^ The XRD patterns of the MnO*_x_*/biochar composites versus unmodified biochar further indicated that there were various forms of MnO*_x_* that appeared on their surfaces (Figure 1b). The peaks at around 35.0°, 40.5°, 58.7°, 70.3°, and 73.8° were indexed to MnO, those at 29.7° and 31.5° were corresponding to Mn_2_O_3_, and that at 50.1° was assigned to MnO_2_.^31−33^ The peak intensities indicated that MnO*_x_* mainly existed as MnO in the produced MnO*_x_*/biochar composites, especially for BioMn600 and BioMn700. The small peaks observed in BioMn400 suggested its relatively poor crystallinity compared to others. The hydrophilicity of the electrodes was measured through the static water contact angles (Figure S4). In comparison with the unmodified biochar electrode (118 ± 3°), all water contact angles of MnO*_x_*/biochar electrodes were less than 90°, suggesting their better hydrophilicity and wettability.^34^ Especially, BioMn600 (56 ± 6°) and BioMn700 (58 ± 3°) showed relatively smaller angles, indicating easy access to the solution and possibly fast mass transfer.^34,35^

**Table 1:** BET Surface Area, Total Pore Volume, and Average Pore Diameter of BioMn400, BioMn500, BioMn600, and BioMn700 Composites

	BET surface area (m^2^ g^–1^)	total pore volume (cm^3^ g^–1^)	average pore diameter (nm)
BioMn400	81.6	0.0406	1.693
BioMn500	105.7	0.0511	1.633
BioMn600	280.8	0.1179	1.879
BioMn700	263.2	0.1121	1.803

The CV curves of MnO*_x_*/biochar electrodes exhibited rectangular-like shapes without discernible redox peaks, suggesting their near-ideal capacitive behaviors (Figure 1c at 5 mV s^–1^ and Figure S5 at 5–100 mV s^–1^).^31,36^ The BioMn600 electrode showed the largest responsive current, indicating the highest specific capacitance of 100.2 F g^–1^, followed by BioMn700 (92.2 F g^–1^), BioMn500 (71.9 F g^–1^), and BioMn400 (51.9 F g^–1^) when the scan rate was 5 mV s^–1^. Dunn’s method (Supporting Information)^37−39^ was employed to reveal the contributions of pseudocapacitance and electrical double layer (EDL) capacitance for the MnO*_x_*/biochar electrodes at the potential of 0.6 V and the scan rate of 5 mV s^–1^ (Figure S6). The total capacitance of MnO*_x_*/biochar electrodes mainly resulted from pseudocapacitance (76–91%) (Figure 1d). The charge–discharge profiles in 30 g L^–1^ NaCl solution further confirmed their ideal capacitive behaviors with linear and symmetric curves (Figure 1e at 0.2 A g^–1^ and Figure S7 at 0.2–2 A g^–1^). Similarly, the BioMn600 electrode had the largest specific capacitance of 103.4 F g^–1^, followed by BioMn700 (95.6 F g^–1^), BioMn500 (86.4 F g^–1^), and BioMn400 (76.2 F g^–1^) at 0.2 A g^–1^ (Figure S8), which were in line with the CV results. The largest specific capacitance of BioMn600 was attributed to the large number of ion adsorption sites that resulted from the high specific surface area.^27,40^ Previous studies have demonstrated that there was a positive correlation between the BET surface area and the specific capacitance of MnO_2_.^27,41^ Additionally, the relatively high percentages of the Mn element in BioMn600 may also result in the large capacitance due to more active sites available for bulk intercalation with cations^28^ (Figure S2). In 300 g L^–1^ NaCl solution, the specific capacitance of the BioMn600 electrode further increased to 183.6 F g^–1^ as the higher electrolyte concentration resulted in faster and easier ion transport (Figure 1e).^42^

The Nyquist plots of MnO*_x_*/biochar electrodes showed a quasi-semicircle in the range of high frequencies followed by a linear tail (Figure 1f). The BioMn600 electrode had the smallest *R*_ct_ value (0.55 Ω), followed by BioMn700 (0.7 Ω), BioMn500 (0.95 Ω), and BioMn400 (1.4 Ω) electrodes. More notably, the *R*_ct_ of the BioMn600 electrode was much lower than those of reported MnO*_x_* electrodes under similar tested conditions,^36,43^ because the carbon matrix of biochar can serve as a conductive network to enhance the electrical contact between MnO*_x_* nanoparticles for fast electron transfer.^28,31^ Similarly, the smallest value of σ was observed for BioMn600 (0.57 Ω s^0.5^) (Figure S9).^44^ The relatively smaller *R*_ct_ and σ values of BioMn600 were due to its larger surface area and better pore properties, which are beneficial for rapid charge transfer and Na^+^ diffusion.

### 3.2 Power Output of Concentration Flow Cells with MnO*_x_*/Biochar Electrodes

**Figure 2:**
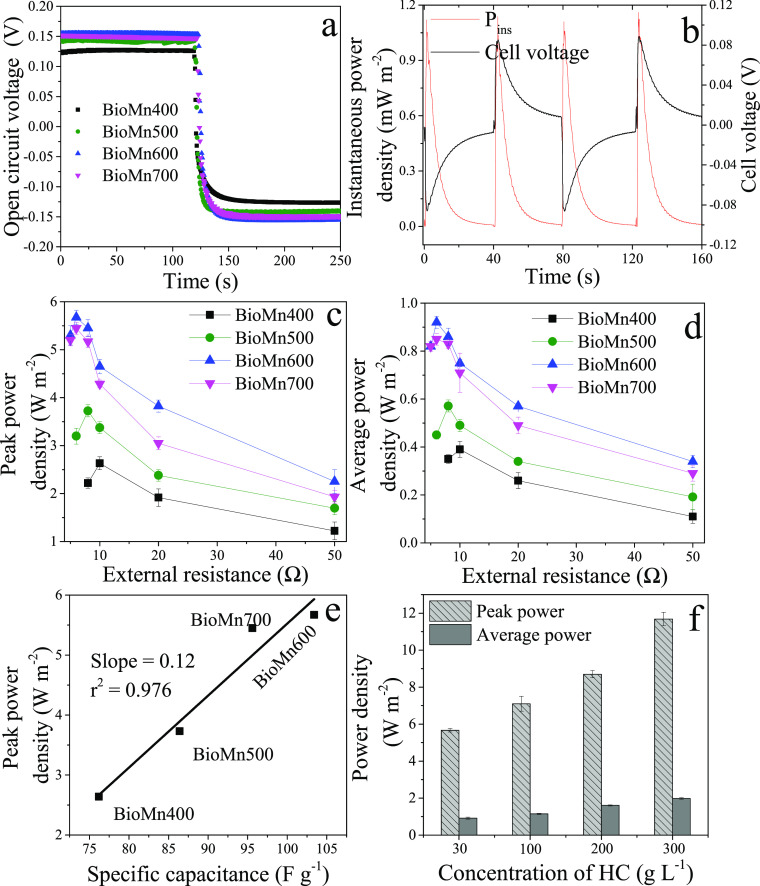
(a) Representative open-circuit voltage (OCV) profiles, (b) representative cell voltage and instantaneous power density of concentration flow cells with BioMn600 electrodes (*R*_ext_ = 20 Ω), (c) peak power density and (d) average power density of concentration flow cells with MnO*_x_*/biochar electrodes at different external resistances fed with 1 g L^–1^ NaCl (LC) and 30 g L^–1^ NaCl (HC) solutions, (e) peak power density produced in the concentration flow cell plotted as a function of specific capacitance (calculated at a current density of 0.2 A g^–1^) for each MnO*_x_*/biochar electrode, and (f) the effect of the concentrations of HC solutions on the power performance of concentration flow cells with BioMn600 electrodes (LC was fixed with 1 g L^–1^ NaCl).

The MnO*_x_*/biochar electrodes were tested in counterflow CFCs fed with 30 and 1 g L^–1^ NaCl solutions (Figure S1). The OCVs of the cells were monitored with the solutions switched at certain time intervals (Figure 2a). The cells with BioMn600 and BioMn700 electrodes had similar and higher OCV of ca. ±0.15 V, followed by those with BioMn500 (ca. ±0.14 V) and BioMn400 (ca. ±0.13 V) electrodes. The OCV of cells resulted from the Donnan potential and the electrode potential (Supporting Information, eq S4). The Donnan potential resulted from the AEM as Cl^–^ moved from the HC to the LC channel, which was ∼0.08 V for all the concentration flow cells.^11^ Therefore, the electrode potential of the cells with BioMn600 and BioMn700 electrodes was 0.07 V, the cell with BioMn500 electrodes was 0.06 V, and the cell with BioMn700 electrodes was 0.05 V. The electrode potential was produced from the activities of Na^+^ with the electrodes via a surface pseudocapacitive reaction (eq 1):^14,27^
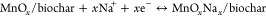
1

When the electrode was immersed in the HC solution, Na^+^ ions intercalated into the electrode. On the other side, Na^+^ ions deintercalated from the electrode to the LC solution. By switching the flow paths of the solutions, the reactions on the electrodes are reversed. This reversible surface pseudocapacitive reaction (eq 1) was confirmed by the XPS high-resolution elemental analysis (Figure S10). Compared to original BioMn600, there was a visible Na 1s peak on the BioMn600 electrode after being discharged in the HC solution, while the Na 1s peak almost disappeared on the BioMn600 electrode after being discharged in the LC solution.

The instantaneous power density and cell voltage increased in the beginning of the cycle and then decreased gradually due to the limited charging capacity of the electrodes. The electrical power output could be obtained again through reversing cell voltages (Figure 2b) when the flow paths were switched in the following cycle. The power output increased first and then decreased when the external resistances changed from 50 Ω to 3 Ω (Figure 2c,d) as the maximum power output is obtained when the external resistance is equal to the internal resistance for electrochemical cells. The cell with BioMn600 electrodes had the largest peak power density of 5.67 W m^–2^ (*R*_ext_ = 6 Ω), followed by BioMn700 (5.45 W m^–2^, *R*_ext_ = 6 Ω), BioMn500 (3.73 W m^–2^, *R*_ext_ = 8 Ω), and BioMn400 (2.64 W m^–2^, *R*_ext_ = 9 Ω) electrodes (Figure 2c). The different optimal *R*_ext_ values suggested that the internal resistances of the cells with MnO*_x_*/biochar electrodes were varied. The cell with BioMn600 electrodes also produced the largest average power density (0.91 W m^–2^) (Figure 2d). The highest power output of the cell with BioMn600 electrodes was due to its low electrode resistances (Figure 1f and Figure S9) and high specific capacitance (Figure 1e and Figure S8). Both the peak power density (Figure 2e) and average power density (Figure S11) were positively correlated with the specific capacitance of MnO*_x_*/biochar electrodes. Similarly, Fortunato *et al*. also reported that the power output of CFCs with different MnO_2_ positively correlated with their respective specific capacitance because high specific capacitance means larger charge storage capacity.^27^ Comparison with the power output of CFCs with biochar electrodes (Bio400–700), which had peak power densities in the range of 0.25–0.45 W m^–2^ (Figure S12), further confirmed that the pseudocapacitance from MnO*_x_* was critical for the high power output of MnO*_x_*/biochar electrodes.

To evaluate the feasibility of SG energy harvest from brine with MnO*_x_*/biochar electrodes, the HC solution was changed from 30 to 300 g L^–1^ NaCl, while the LC solution was kept at 1 g L^–1^ NaCl in the cell with BioMn600 electrodes. The power outputs increased concurrently with increasing concentrations of HC solutions (Figure 2f). When the HC solution was 300 g L^–1^ NaCl, the peak power density was up to 11.68 W m^–2^ and the average power density was 1.98 W m^–2^ (Figure 2f). This increment was due to the higher gained cell voltage with high salinity gradients (Figure S13), larger specific capacitance (Figure 1e), and lower solution resistance with higher concentration solutions. It is demonstrated that CFCs with MnO*_x_*/biochar electrodes can be used for SG energy harvest from brines.

**Figure 3:**
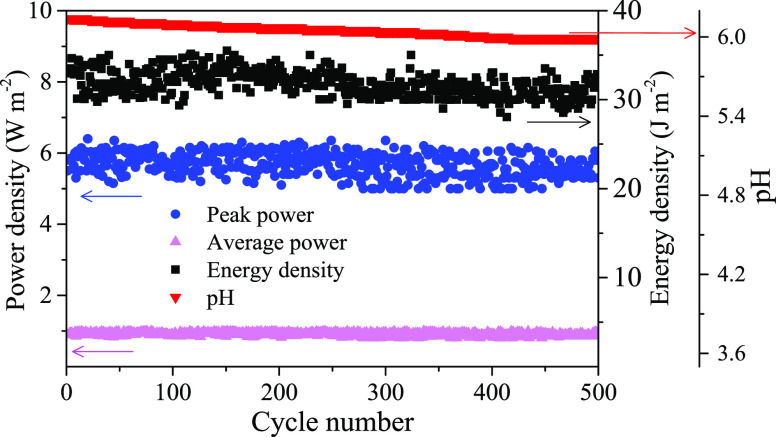
Peak power density, average power density, energy density, and outflow pH recorded for 500 cycles of concentration flow cells with BioMn600 electrodes under an optimum external resistance of 6 Ω fed with 1 g L^–1^ NaCl (LC) and 30 g L^–1^ NaCl (HC) solutions.

To test the cycling performance, the cell with BioMn600 electrodes fed with 30 and 1 g L^–1^ NaCl solutions was run for 500 consecutive cycles (Figure 3). The performance of the cell was very stable with the peak power density stabilized around 5.67 W m^–2^, the average power density remained at ∼0.91 W m^–2^, and the energy density stayed at ∼32.7 J m^–2^. In addition, the pH of the outflow only slightly changed from 6.14 to 5.99 over the 500 cycles without obvious fluctuations (Figure 3). Under the current density of 1 A g^–1^, the specific capacitance retention of the BioMn600 electrode was 97% after 1000 charge–discharge cycles (Figure S14). The high stability of the BioMn600 electrode was another reason for the excellent cyclability of the cell. Similarly, Cheng *et al*.^26^ reported that the biochar@MnO hybrid can deliver an excellent reversible capacity for over 500 cycles in lithium batteries. The biochar can work as a supportive backbone in MnO*_x_*/biochar composites, which can significantly reduce the particle aggregation and buffer volume contraction–expansion of manganese oxides and thus ensure the structure integrity and give long-term stability.^19,26^ Moreover, like other carbon/MnO*_x_* hybrids, the MnO*_x_*/biochar composite can obtain good corrosion resistance and better electronic conductivity, which also favor for the cycling stability.^31^

More notably, the power output of CFCs with BioMn600 electrodes (peak power density = 5.67 W m^–2^; average power density = 0.91 W m^–2^) was higher than previous CFCs with various MnO_2_ electrodes (peak power density, <4 W m^–2^; average power density, <0.81 W m^–2^)^27^ and most other electrodes (i.e., BiCl_3_, BiOCl, MoS_2_, and carbonized peat moss)^13−15,27^ (Table S1). The reason could be due to the synergistic effects between biochar and MnO*_x_*. The carbon-rich porous network of the MnO*_x_*/biochar hybrid can enhance its conductive connectivity, thereby reducing the electrode resistance (as proved by the small *R*_ct_ and σ values, Figure 1f and Figure S9).^26,43^ On the other hand, the MnO*_x_* nanoparticles embedded in the carbon matrix of biochar can increase the active sites and improve the specific capacitance, thereby having fast pseudocapacitive reactions with Na^+^ ions (eq 1).^45^ Previous studies also demonstrated that the carbon/MnO*_x_* hybrid can deliver better performance in a supercapacitor^28^ and capacitive deionization.^43^ Though the power densities of BioMn600 electrodes were lower than the cell with CuHCF electrodes,^11^ the MnO*_x_*/biochar composite was made from agriculture residues (rice husk) that can also be replaced by other kinds of renewable biomass wastes (i.e., forestry wastes and livestock wastes) with almost zero cost. Biochar even favors for waste management and greenhouse gas mitigation^19^ and can earn credits if traded in the global carbon market.^46^ Moreover, partial dissolution of CuHCF^11^ was observed in the long-term cycling investigation, which can cause environment problems as both Cu^2+^ and cyanide ions are toxic to aquatic life, while the biochar and MnO*_x_* were environmentally benign and the MnO*_x_*/biochar composite was very stable as demonstrated before due to interactions between MnO*_x_* and biochar. Additionally, the synthesis method of MnO*_x_*/biochar composites by pyrolyzing rice husks soaked with KMnO_4_ is very simple, which can enable easy industrial-scale production of electrode materials without any environmental issues.

### 3.3 Effects of Inorganic Ions on the Power Output of Concentration Flow Cells with MnO*_x_*/Biochar Electrodes

**Figure 4:**
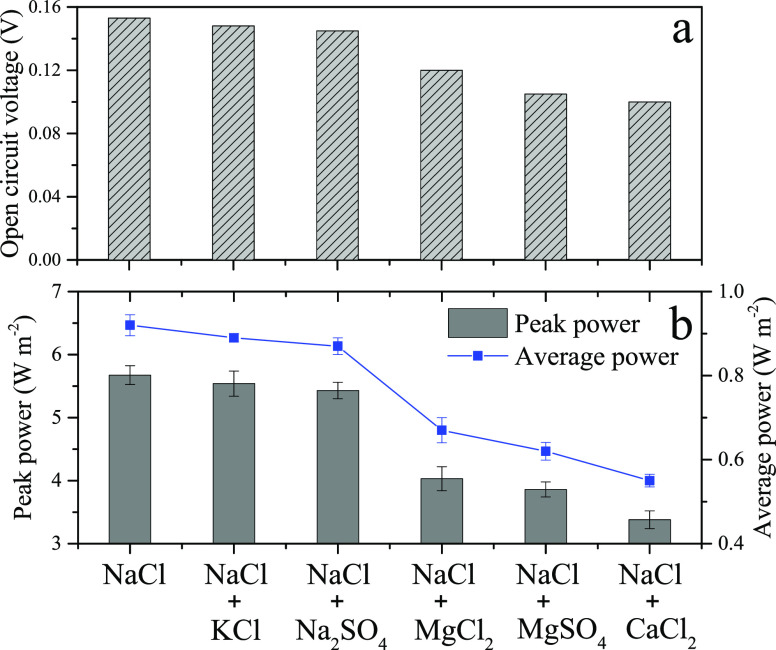
(a) Open-circuit voltage and (b) peak power density and average power density of the concentration flow cell with BioMn600 electrodes under different ionic compositions in the feed solutions.

**Figure 5:**
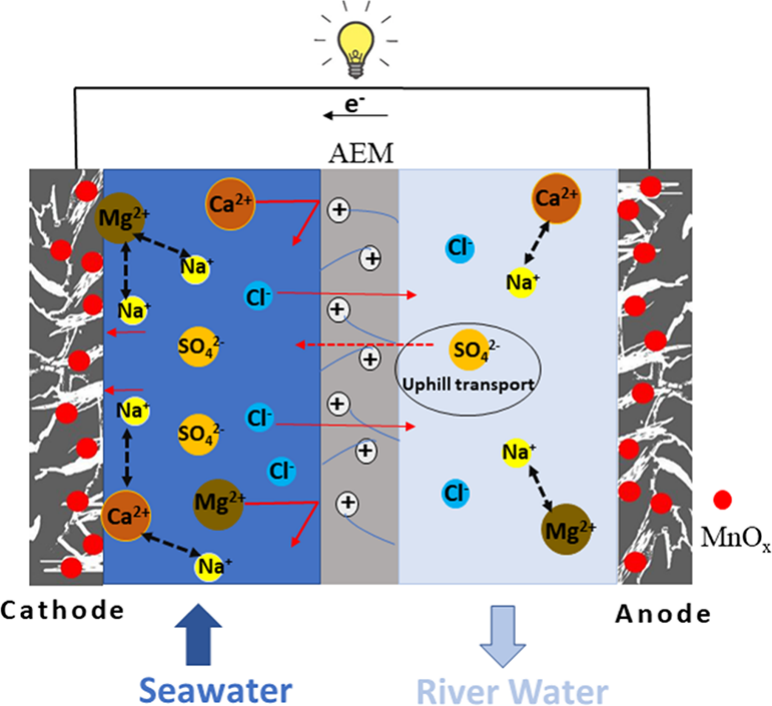
Schematic illustration of multivalent ions’ (Ca^2+^, Mg^2+^, and SO_4_^2–^) influences on the concentration flow cell with BioMn600 electrodes.

The influences of inorganic ions on the power output were investigated in CFCs with BioMn600 electrodes. As 10% (in molar) of NaCl was replaced by other inorganic salts (KCl, Na_2_SO_4_, MgCl_2_, MgSO_4_, or CaCl_2_), the OCVs of the cells changed significantly (Figure 4a). The trend of the negative impact of inorganic ions on the OCV relative to pure NaCl was CaCl_2_ > MgSO_4_ > MgCl_2_ > Na_2_SO_4_ > KCl. The power output also changed in a similar pattern (Figure 4b). When 10% of NaCl was replaced by KCl, the peak power density (5.54 W m^–2^) only decreased by 2.3% compared to 100% NaCl, suggesting that the monovalent K^+^ had no obvious negative impact on the power output. When replaced by Na_2_SO_4_, the peak power density (5.43 W m^–2^) decreased by 4.23%, probably because SO_4_^2–^ has a large hydrated radius, which can cover the charges fixed on the AEM or trapped inside the membrane, thereby decreasing the membrane permeability.^47,48^ Another reason could be due to the uphill transport effect. To obey the electroneutrality on both sides of the AEM, SO_4_^2–^ could be uphill transported from LC to HC solutions (Figure 5) as the electromotive force of Cl^–^ was higher than that of SO_4_^2–^, similar to the uphill transport that occurred across the cation-exchange membrane (CEM) for Mg^2+^ and Na^+^ in RED systems.^48,49^ The uphill transport of SO_4_^2–^ could sacrifice the salinity difference of Cl^–^, while no net charge was transported at the same time and therefore reduced the power output.^49^

The Mg^2+^ and Ca^2+^ had more pronounced negative effects on the OCV and power output of the cells. With the addition of MgCl_2_, the OCV decreased to 0.12 V and further declined to ∼0.1 V with MgSO_4_ and CaCl_2_ (Figure 4a). Theoretically, the positive Mg^2+^ and Ca^2+^ ions would not affect the membrane potential as the AEM is positively charged.^18,50^ Therefore, the reduced OCV in the presence of Mg^2+^ and Ca^2+^ should result from the decrement of electrode potentials due to inorganic fouling as Na^+^ (3.58 Å for hydrated radius) near the electrode surface could be replaced by multivalent ions that have larger hydrated radii (4.28 Å for Mg^2+^ and 4.12 Å for Ca^2+^) (Figure 5).^51^ Mg^2+^ and Ca^2+^ ions could more easily migrate to the surface of the electrodes, which had negative surface charges as indicated by the negative zeta potential of BioMn600 (Figure S15).^52^ As the pore size of BioMn600 (1.88 nm) was much larger than those studied ions, Mg^2+^ and Ca^2+^ can accumulate in the pores of electrodes, resulting in pore blockage and repulsion force toward Na^+^,^53^ and consequently lower the electrode potentials. This is consistent with a previous study on CapMix based on EDLs that the electrode potential of carbon electrodes deteriorated under the presence of Mg^2+^ or Ca^2+^.^54^ Correspondingly, the peak power density decreased by 28.9% in the presence of MgCl_2_ (4.02 W m^–2^) and further decreased by 31.9% in the presence of MgSO_4_ (3.86 W m^–2^) compared to 100% NaCl. The Ca^2+^ exerted more negative impacts on the power output than Mg^2+^. The peak power density (3.38 W m^–2^) decreased by 40.1% in the presence of CaCl_2_. This could be due to the relatively larger diffusion coefficient of Ca^2+^ (0.79 × 10^–9^ m^2^ s^–1^) than Mg^2+^ (0.71 × 10^–9^ m^2^ s^–1^),^51^ which would result in more negative effects on the activity and transfer of Na^+^.

Another reason for the decrement of power output in the presence of inorganic ions could be the decreased specific capacitances. Compared to the pure NaCl (100.2 F g^–1^), the specific capacitance of BioMn600 electrodes reduced to 95.9 F g^–1^ in the solution with the addition of KCl, followed by Na_2_SO_4_ (87.8 F g^–1^), MgCl_2_ (84.6 F g^–1^), CaCl_2_ (82.8 F g^–1^), and MgSO_4_ (82.6 F g^–1^) due to the steric repulsion on the surface of BioMn600^54^ (Figure S16). Additionally, previous studies indicated that the existence of multivalent ions (Ca^2+^, Mg^2+^, and SO_4_^2–^) can increase the membrane resistance and non-ohmic resistance,^55,56^ which in turn reduced the power output of RED cells.^47,57^ In all, in line with the OCV, the trend of negative impacts of inorganic ions on the power output of CFCs with BioMn600 electrodes was CaCl_2_ > MgSO_4_ > MgCl_2_ > Na_2_SO_4_ > KCl. Although more studies are still needed, our results clearly demonstrated that multivalent ions (Ca^2+^, Mg^2+^, and SO_4_^2–^) had obvious negative impacts on the power output of CFCs. When using natural waters (e.g., river water and seawater), some measurements need to be taken to mitigate the negative impacts from multivalent ions, such as employment of monovalent ion-selective membranes and softening of the feed waters.^18^

## 4 Conclusions

The easily synthesized, facile industrial-scale processing, environmentally friendly, low cost, and robust MnO*_x_*/biochar composite can be used in CFCs for efficient SG energy harvest. The peak power density was 5.67 W m^–2^ (ave. = 0.91 W m^–2^) using 1 and 30 g L^–1^ NaCl as the feed solutions attributed to the high specific capacitances and low electrode resistances of MnO*_x_*/biochar electrodes, which were higher than all previously reported MnO_2_ electrodes. Additionally, the effects of inorganic ions on the power output of CFCs were investigated for the first time. The monovalent ion (K^+^) showed no obvious effect on the power output, while the bivalent ions (Mg^2+^ and Ca^2+^) greatly reduced the power density by 30–40% compared to pure NaCl solutions probably due to the decreased electrode potentials and specific capacitances.

## Supporting Information Available

Characterization methods of MnO*_x_*/biochar electrodes; theory of CFCs; Dunn’s method; summary of power performance of CapMix and CFCs with other kinds of electrodes; characteristics of studied ions; schematic of the counterflow CFC; SEM–EDS, FTIR, and water contact angle of MnO*_x_*/biochar composite; CVs at scan rates of 5–100 mV s^–1^; specific capacitances and charge/discharge profiles of MnO*_x_*/biochar electrodes at the current densities of 0.2–2 A g^–1^; EIS measurements of MnO*_x_*/biochar electrodes; XPS spectra for Na 1s of BioMn600 electrodes; the correlation analysis between specific capacitance and power density; power performance of CFCs with biochar electrodes; open-circuit voltage of CFCs with BioMn600 electrodes under brine; cycling stability of BioMn600 electrodes over 1000 cycles; CV curves and specific capacitance for the BioMn600 electrode under different ionic compositions (PDF)
