# Supplementary material for: Facile Designed Manganese Oxide/Biochar for Efficient Salinity Gradient Energy Recovery in Concentration Flow Cells and Influences of Mono/Multivalent Ions
Source: ACS Appl Mater Interfaces. 2021 Apr 23;13(17):19855–63. doi: 10.1021/acsami.0c21956 (PMC8288956; doi:10.1021/acsami.0c21956)
Supplement: Supplementary file 1 — am0c21956_si_001.pdf [file am0c21956_si_001.pdf]

Supporting Information for

Facile Designed Manganese Oxide/Biochar for Efficient Salinity Gradient Energy Recovery in Concentration Flow Cells and Influences of Mono(multi)valent Ions

Guangcai Tan[a,b], Nan Xu[c], Dingxue Gao[c], Xiuping Zhu[b,*]

[a] *CAS Key Laboratory of Urban Pollutant Conversion, Department of Environmental Science and Engineering, University of Science and Technology of China, Hefei, 230026, China*

[b] *Department of Civil and Environmental Engineering, Louisiana State University, Baton Rouge, LA 70803, USA*

[c] *Shenzhen Engineering Research Center for Nanoporous Water Treatment Materials, School of Environment and Energy, Peking University Shenzhen Graduate School, Shenzhen 518055, China*

[*] *Corresponding author. Tel./Fax: +1-(225) 578-1523. E-mail address: xzhu@lsu.edu.*

Pages: 25

Figures: 16

Tables: 2

Electrode Characterizations

Specific surface areas and porosities were measured by a Brunauer– Emmett–Teller (BET) N_2 adsorption method (ASAP 2020 PLUS, Micromeritics, US). A scanning electron microscope (SEM, JSM-6610 LV, Japan) coupled with an energy dispersive X-ray spectroscopy (EDS, Oxford Instruments, USA) was used to examine the morphology and elemental composition of MnO_x/biochar composites. X-ray diffractometer (Empyrean, Malvern Panalytical, UK) with Cu Ka radiation was used for X-ray Diffraction (XRD) analysis. The interlayer spacing was obtained using Bragg equation from the peak center (at $2\theta \sim 40°$). X-ray photoelectron spectroscopy (XPS) was conducted on an AXIS165 spectrometer (ESCA 2SR, Scienta Omicron, US) using a twin-anode Al Ka radiation as the X-ray source. Zeta potentials were obtained by a zeta potential analyzer (Zetasizer Nano, UK). The water-contact angles of the electrodes were obtained by an optical contact angle goniometer (KSV, Finland), and the data were an average of three replicate measurements.

Electrochemical characterizations of biochar/MnO_x electrodes were performed using a potentiostat (VMP3, Bio-Logic) in a single-chamber cell with a platinum-coated titanium counter electrode and an Ag/AgCl reference electrode. Cyclic voltammetry (CV) was scanned from 0 V to 0.6 V at a rate of 5-100 mV s^{-1} in HC (30 g L^{-1} NaCl) solution. The specific capacitance (C_s, F g^{-1}) of the electrode can be calculated from the area of the voltammograms by the means of the following formula (Eq. S1):

$$C_s = \frac{\oint I \, dV}{\Delta V \cdot m \cdot v} \qquad \text{(S1)}$$

where I (A), ΔV (V), m (g), and v (V s^{-1}) are current, voltage window, mass of active materials in working electrode, and the scan rate, respectively.

Electrochemical impedance spectroscopy (EIS) measurements were performed with a perturbation amplitude of 10 mV around the equilibrium potential (OCV). The data were collected in the frequency range from 100 kHz to 10 mHz. The NaCl solutions with a concentration of 30 g L^{-1} were used. Galvanostatic charge/discharge tests were conducted in a potential range of 0 – 0.6 V at current densities of 0.2-2 A g^{-1}. The specific capacitance (C_s, F g^{-1}) of electrodes from galvanostatic charge/discharge was then calculated by Eq. (S2).

$$C_s = \frac{I \cdot \Delta t}{\Delta V \cdot m} \quad \text{(S2)}$$

where I is the charge–discharge current (A), ΔV is the potential window (V), Δt is the discharge time (s), and m is the mass of active materials in working electrode (g).

Theory of concentration flow cell

The maximum power density (P_{max}, W m^{-2}) obtainable from the concentration flow cell depends on the OCV (V) and the total resistance (R_{tot}, Ω) according to:

$$P_{max} = \frac{OCV_{tot}^2}{A\,R_{tot}} \quad \text{(S3)}$$

where A (m^2) is the working area of the anion-exchange membrane. The equation is equivalent to the Maximum Power Transfer theorem, where the power has been divided by the working membrane area to obtain units of W m^{-2}.

The total OCV of the concentration flow cell arises from the Donan potential from AEM (OCV_m) and electrode potential (OCV_e):

$$OCV_{tot} = OCV_m + OCV_e \quad \text{(S4)}$$

For the single NaCl solution, the potential of AEM (OCV_m, V) can be calculated using the Nernst equation:[1,2]

$$OCV_m = \alpha \frac{RT}{z_1 F} \ln \frac{C_{HC}^{Cl^-}\,\gamma_{HC}^{Cl^-}}{C_{LC}^{Cl^-}\,\gamma_{LC}^{Cl^-}} \quad \text{(S5)}$$

where α is the permselectivity of the membrane, R is the universal gas constant (8.314 J mol^{-1} K^{-1}), T is absolute temperature (298 K in our experiments), z_1 is the valence of the ionic specie Cl^-, F is the Faraday constant (96 485 C mol^{-1}), C (mol L^{-1}) and γ (dimensionless) are the concentration and activity coefficients of Cl^-, respectively.

When the HC and LC containing NaCl + $MgSO_4$ or Na_2SO_4:

$$OCV_m = \alpha\frac{RT}{F}\left(\frac{1}{z_1}\ln\frac{C_{HC}^{Cl^-}\gamma_{HC}^{Cl^-}}{C_{LC}^{Cl^-}\gamma_{LC}^{Cl^-}} + \frac{1}{z_2}\ln\frac{C_{HC}^{SO_4^{2-}}\gamma_{HC}^{SO_4^{2-}}}{C_{LC}^{SO_4^{2-}}\gamma_{LC}^{SO_4^{2-}}}\right) \qquad (S6)$$

where z_2 is the valence of the ionic specie SO_4^{2-}, C and γ represent the concentration and activity coefficient of each ion in HC and LC, respectively.

According to previous studies,[3,4] the electrode potential is based on the acitivity of Na^+, and can be written as:

$$OCV_e = \frac{RT}{F}\ln\left(\frac{a_{Na^+,\mathrm{HC}}}{a_{Na^+,\mathrm{LC}}}\right) \qquad (S7)$$

where $a_{Na^+,\mathrm{HC}}$ and $a_{Na^+,\mathrm{LC}}$ are the acitvity of Na^+ in HC and LC, respectively.

The total resistance of the concentration flow cell characterizes the amount of energy lost due to the interaction among the ions, AEM and electrodes. Total stack resistance can be expressed as:

$$R_{tot} = R_{HC} + R_{LC} + R_m + R_{non\text{-}ohmic} \qquad (S8)$$

where R_{HC} and R_{LC} represent the resistances of HC and LC compartments, respectively; R_m is the ion exchange membrane resistance, and $R_{non\text{-}ohmic}$ is the non-ohmic resistance which could be resulted from the resistance of the diffusion boundary layer.[5]

Dunn's method

The quantity analysis of the capacitance contributions from the surface capacitive effects (EDL) and diffusion controlled process (pseudocapacitance) is enabled by Dunn's method.[6-8]

The current density (i) from the CVs can be expressed as the following two parts, k_1v and $k_2v^{0.5}$, at a fixed potential:

$$i = k_1v + k_2v^{0.5} \qquad \text{(S9)}$$

where the first term k_1v accounts for the current density contributed from the EDL capacitive effects while the second term $k_2v^{0.5}$ is the current density associated with the pseudocapacitive reactions. The equation can be rearranged by dividing the $v^{0.5}$ on both sides:

$$iv^{-0.5} = k_1v^{0.5} + k_2 \qquad \text{(S10)}$$

Thus, by reading i from the CVs at a series of scan rates and then plotting $iv^{-0.5}$ vs. $v^{0.5}$, one expects to obtain a linear fitting line with a slope of k_1 and a y-intercept of k_2. Fig. S6 gives the results of $iv^{-0.5}$ vs. $v^{0.5}$ plot collected for MnO_x/biochar electrodes at a potential of 0.6 V. Using the k_1 and k_2 values in Eq. (S10) allows one to differentiate the capacitance contribution from EDL and pseudocapacitance at the specific potential V and a selected scan rate, v.

Table S1. Comparison with other electrochemical systems for salinity gradient energy harvest.

	Electrode	Salinity (HC/LC) (g L^{-1})	Membrane	Power density (W m^{-2})	Ref. & year
CapMix	AC/AC [b]	30/1	AEM/CEM	0.2	9 (2012)
	$Na_{2-x}Mn_5O_{10}$/AgCl	87.8/1.7	No	0.105	10 (2011)
	$Na_4Mn_9O_{18}$/AC	35.1/0.058	AEM	0.097	11 (2017)
	AC/AC	30/1	AEM/CEM	0.095	12 (2013)
	AC/AC	29.84/0.58	AEM/CEM	0.072	13 (2010)
	CuHCF/BiOCl	30/1	No	0.1	14 (2019)
	AC/AC	80/4	AEM/CEM	0.16	15 (2020)
CFC	CuHCF/CuHCF	30/1	Filtration membrane	0.411	16 (2016)
	CuHCF/CuHCF	30/1	AEM	12.6	3 (2017)
	$BiCl_3$/$BiCl_3$	30/1	CEM	3.17	17 (2018)
	Carbonized peat moss /Carbonized peat moss	30/1	CEM	5.33	18 (2018)
	BiOCl/BiOCl	30/1	CEM	4.36	19 (2019)
	MoS_2/ MoS_2	30/1	AEM	5.21	20 (2020)
	MnO_2/MnO_2	30/1	AEM	4	4 (2020)
	BioMn600/BioMn600	30/1	AEM	5.67	This study

Note: a. --- unknown

b. AC = activated carbon

c. AEM = anion-exchange membrane

d. CEM = cation-exchange membrane

Table S2 Characteristics of various ions used in this study.[21-24]

Ion	Molar conductivity (10^{-4} S m^2 mol^{-1})	Diffusion coefficient (m^2 s^{-1})	Ionic radius (Å)	Radius of hydrated ion (Å)
Na^+	50.08	1.33×10^{-9}	0.98	3.58
Cl^-	76.31	2.03×10^{-9}	0.181	3.31
K^+	73.48	1.96×10^{-9}	0.133	3.32
Mg^{2+}	53	0.71×10^{-9}	0.66	4.28
Ca^{2+}	59.47	0.79×10^{-9}	0.99	4.12
SO_4^{2-}	80	1.07×10^{-9}	0.22	3.79

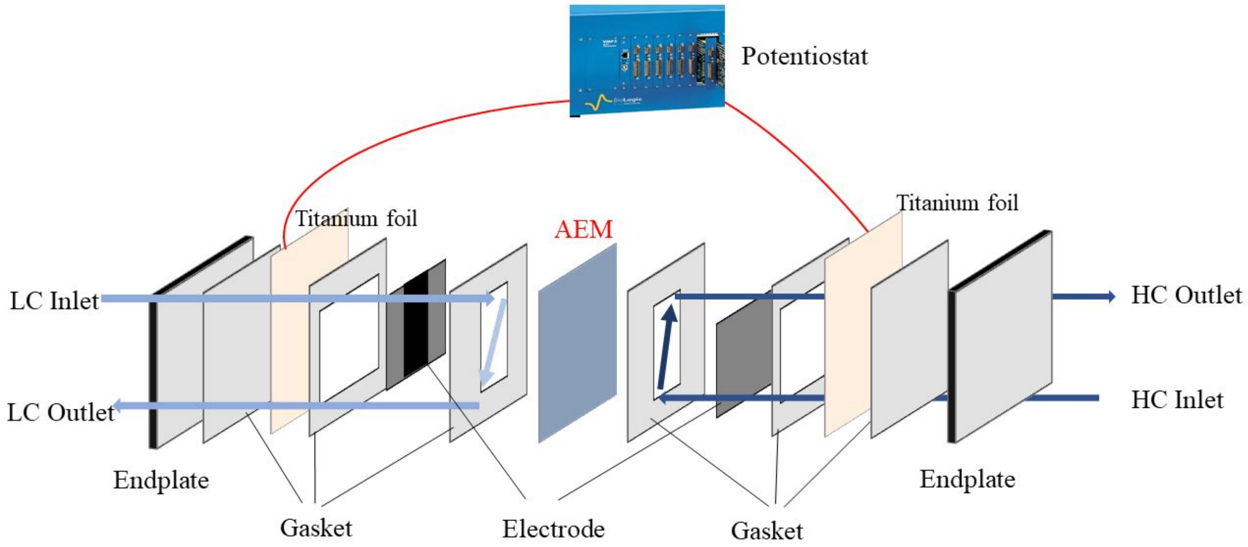

Figure. S1. A schematic of a custom-built counter-flow concentration flow cell.

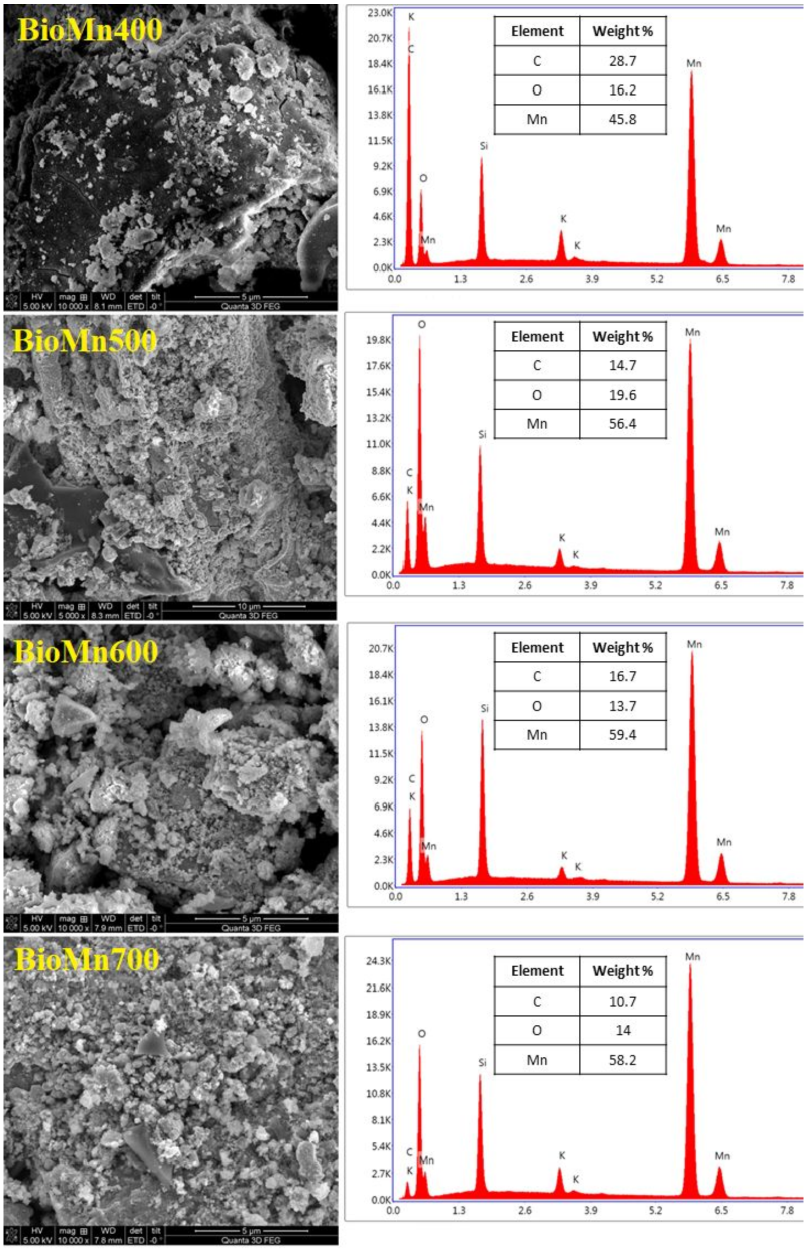

Figure. S2 Scanning electron microscopy images (left) and corresponding energy dispersive X-ray spectra (right) of BioMn400, BioMn500, BioMn600 and BioMn700.

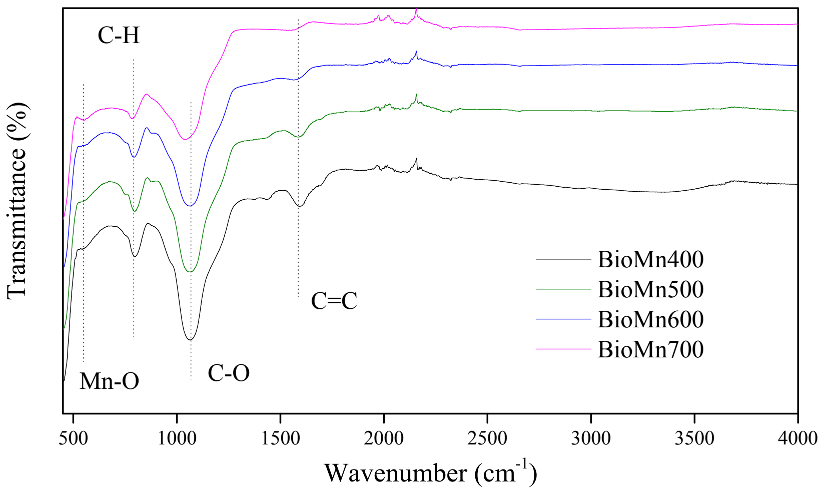

Figure. S3. FTIR spectra of BioMn400, BioMn500, BioMn600 and BioMn700.

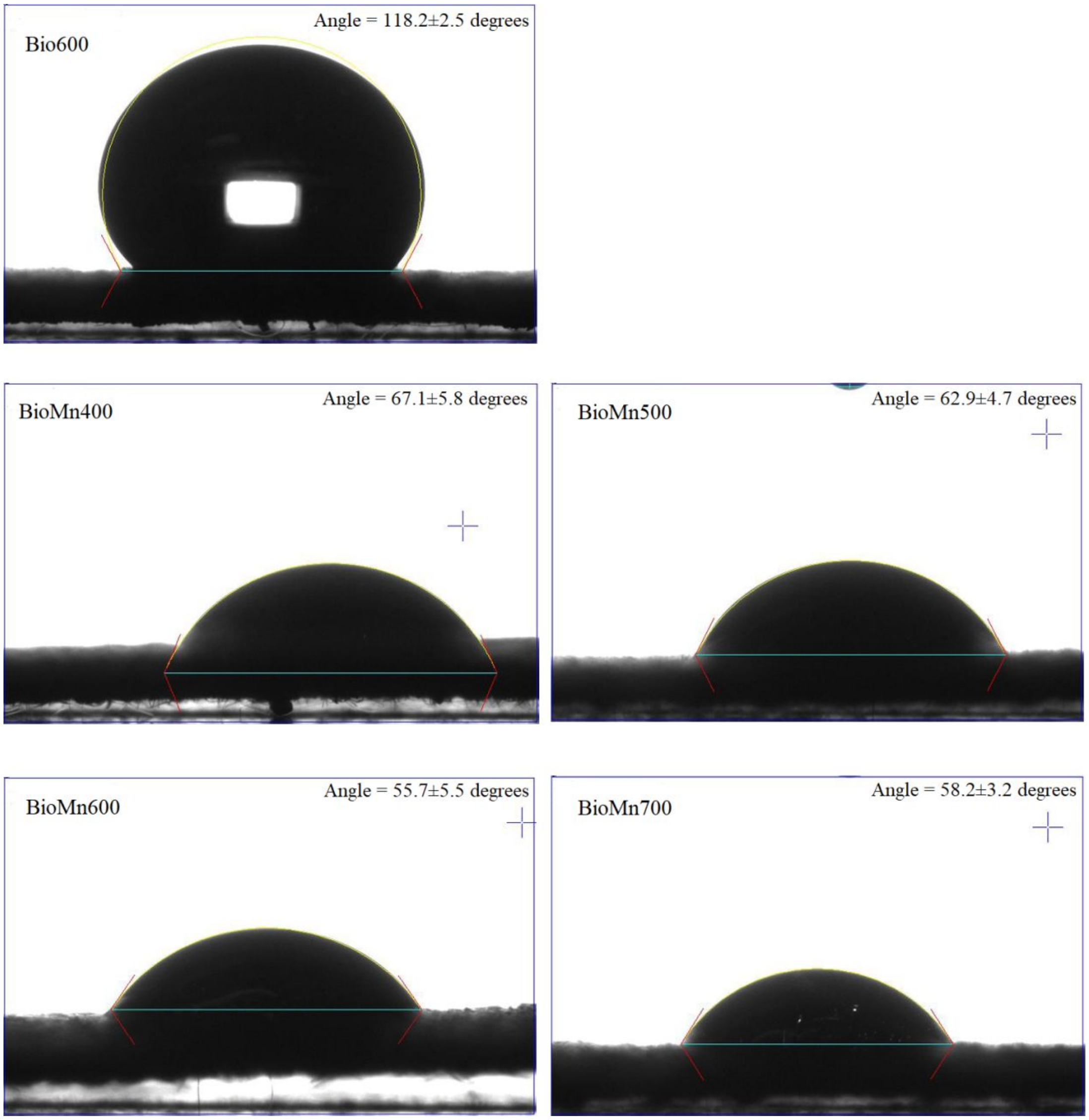

Figure. S4. Water-contact angle measurements for Bio600, BioMn400, BioMn500, BioMn600 and BioMn700 electrodes.

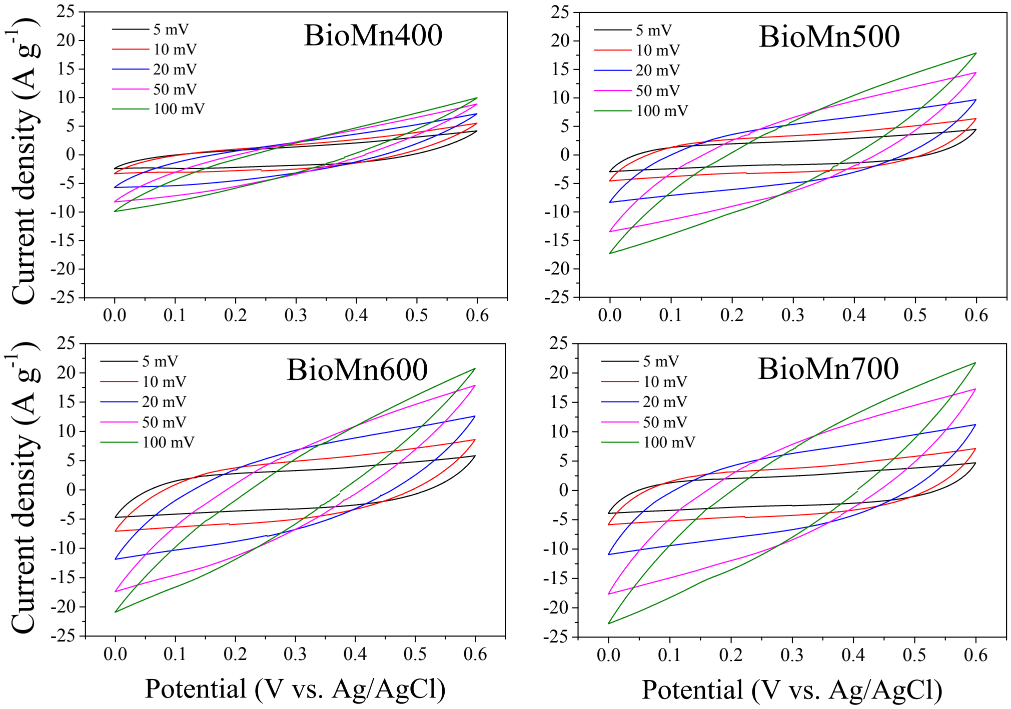

Figure. S5. Cyclic voltammograms of BioMn400, BioMn500, BioMn600 and BioMn700 electrodes at scan rates of 5-100 mV s^{-1}.

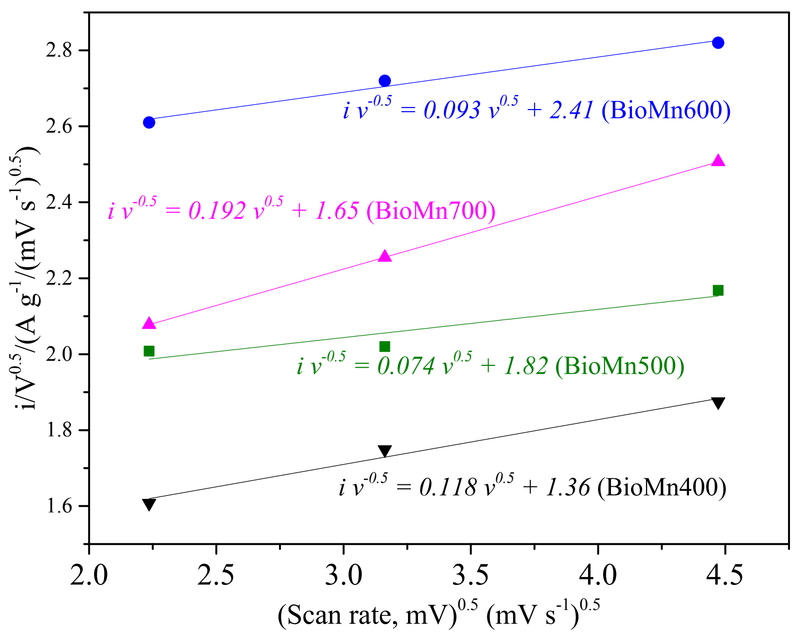

Figure. S6. Dunn's method analysis of $i\ v^{-0.5}$ vs. $v^{0.5}$ plot for the MnO_x/biochar electrodes at a potential of 0.6 V vs. Ag/AgCl.

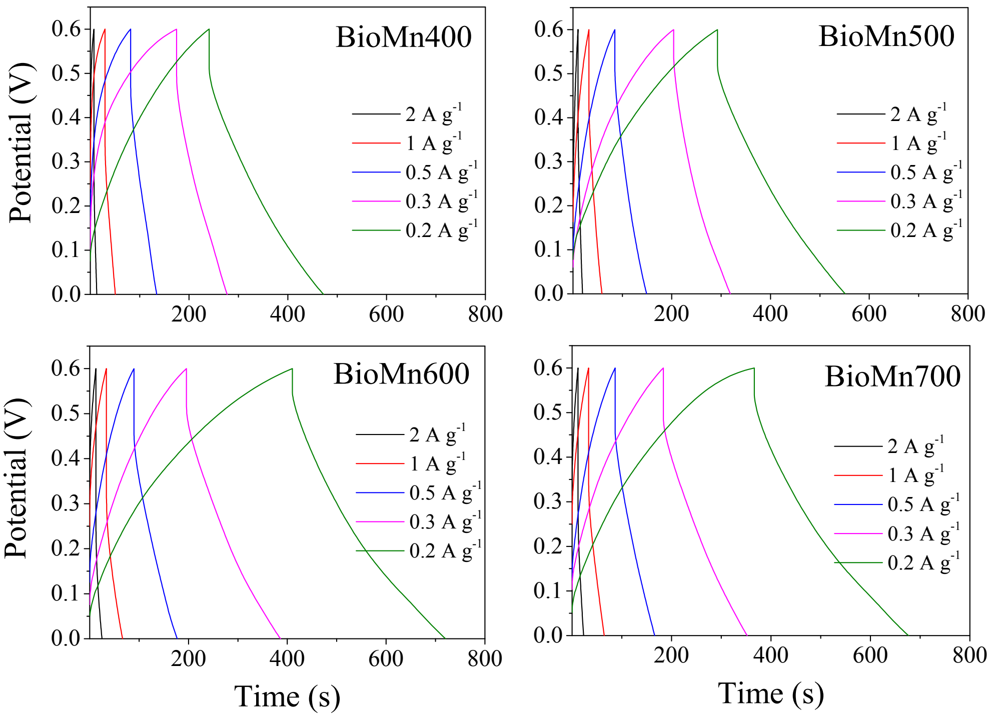

Figure. S7. Galvanostatic charge/discharge profiles of BioMn400, BioMn500, BioMn600 and BioMn700 electrodes at current densities of 0.2-2 A g^{-1}.

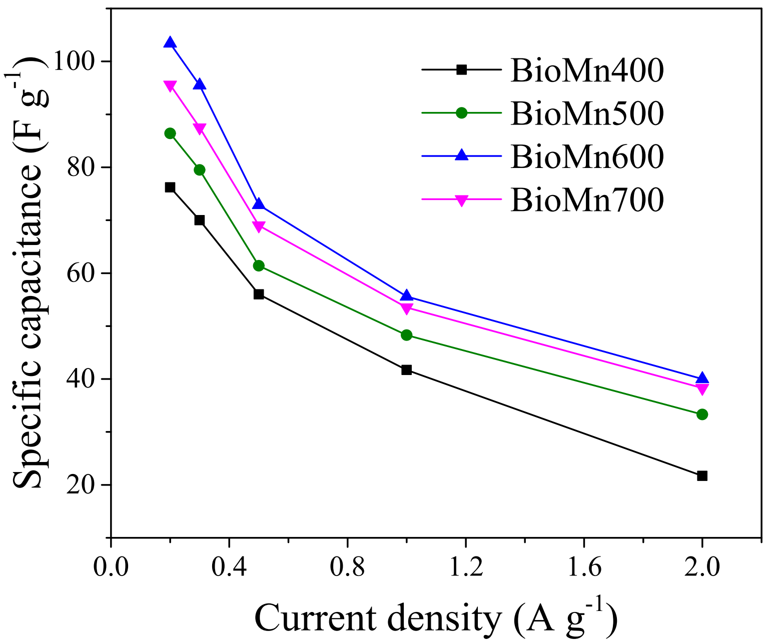

Figure. S8. Specific capacitances of BioMn400, BioMn500, BioMn600 and BioMn700 electrodes at different current densities.

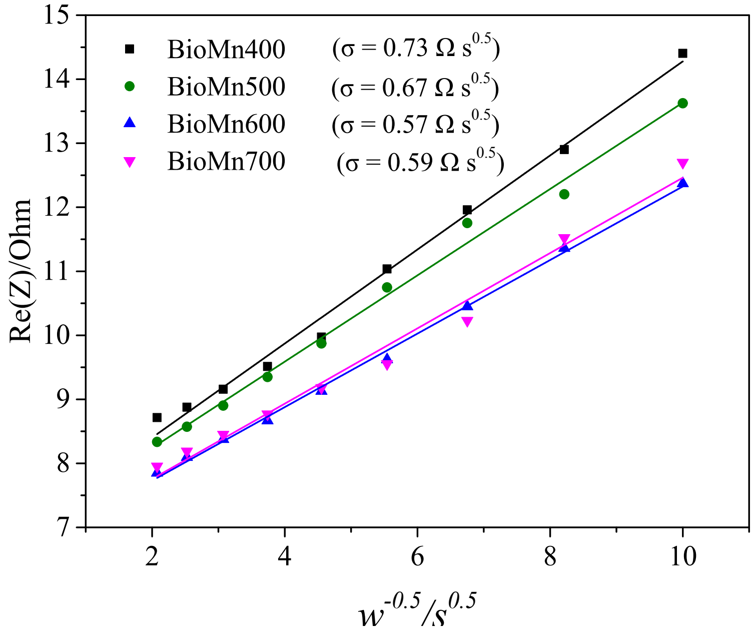

Figure. S9. Re(Z) *vs*. the reciprocal of the square root of frequency ($w^{-0.5}$) in the intermediate to low frequency range (0.01-0.2 Hz) for the BioMn400, BioMn500, BioMn600 and BioMn700 electrodes at the NaCl concentration of 30 g L^{-1}

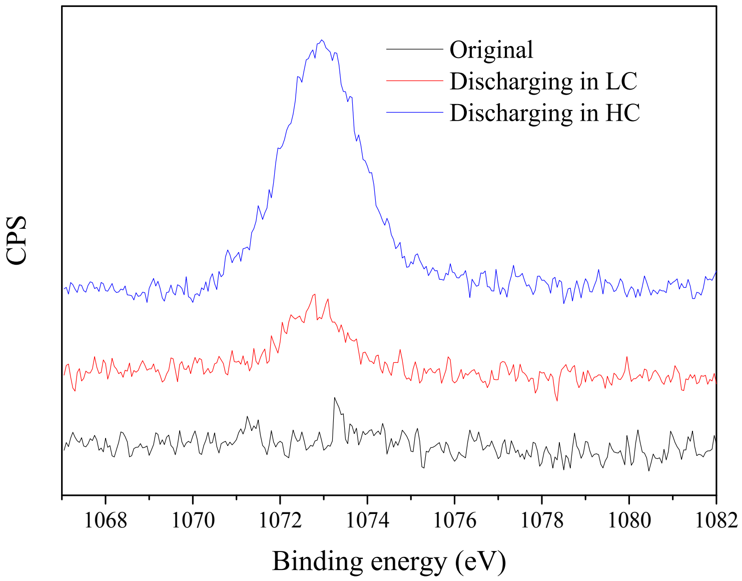

Figure. S10. XPS high resolution elemental analysis showing characteristic Na 1s peak for the original BioMn600 electrode and the electrodes discharging in 30 g L^{-1} and 1 g L^{-1} NaCl solutions.

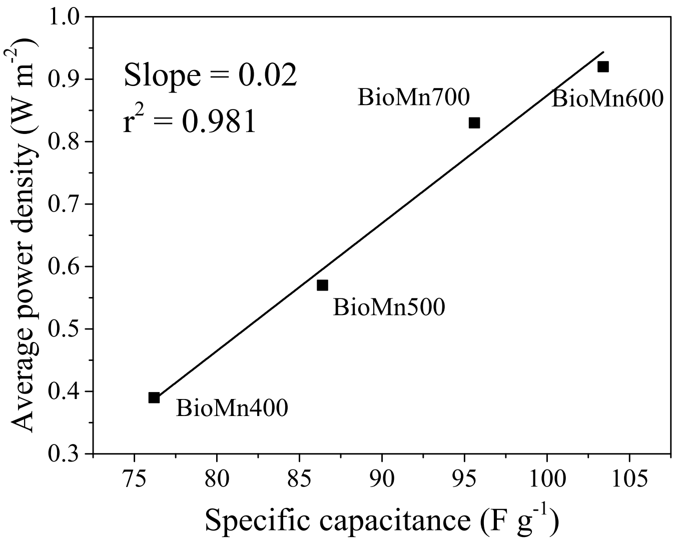

Figure. S11. Average power density produced in the concentration flow cell plotted as a function of specific capacitance for each MnO_x/biochar (BioMn400, BioMn500, BioMn600 and BioMn700 electrodes) electrode. Specific capacities were calculated from at a current density of 0.2 A g^{-1}.

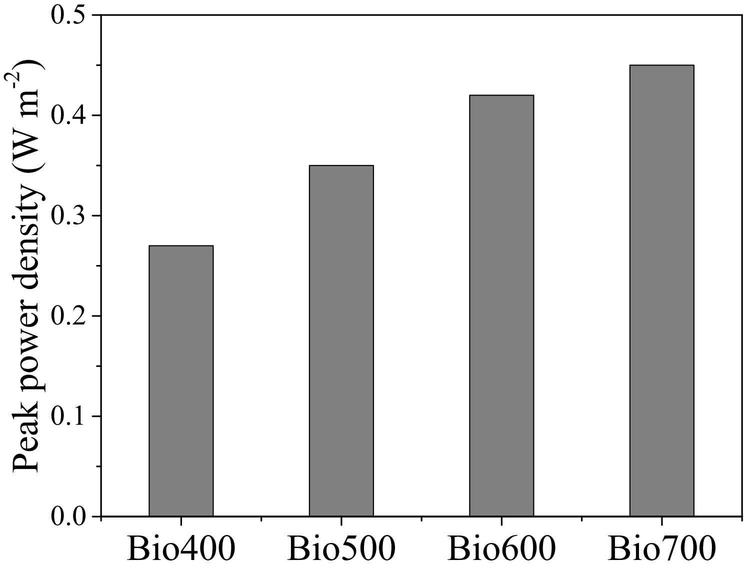

Figure. S12. Peak power density of the concentration flow cell with different biochar electrodes.

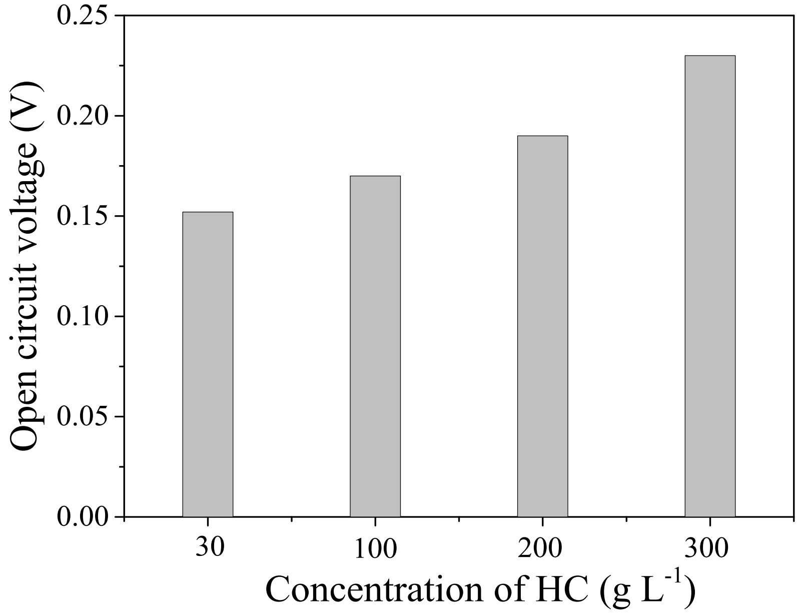

Figure. S13. The effect of the concentration of HC solutions (30, 100, 200 and 300 g L^{-1}) on the open circuit voltage of concentration flow cells with BioMn600 electrodes (LC was fixed with 1 g L^{-1} NaCl).

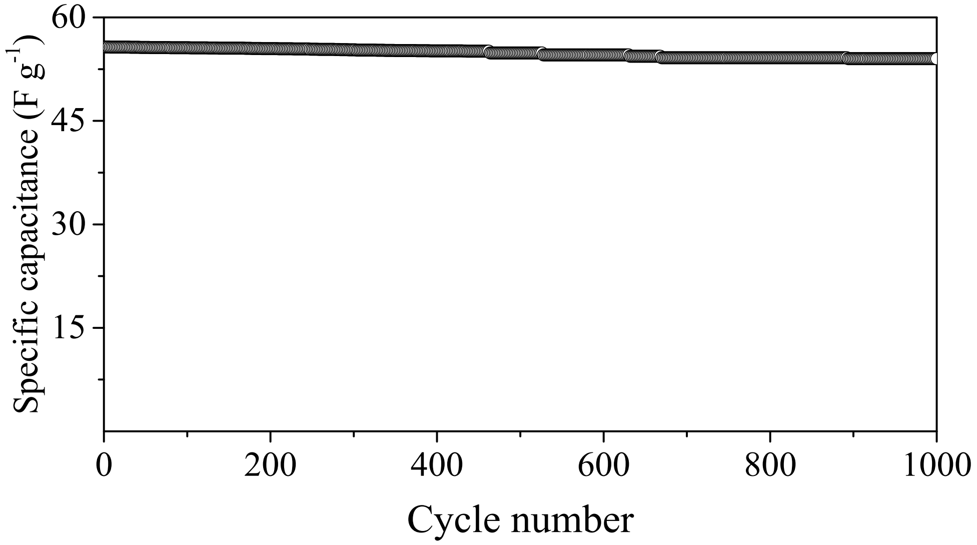

Figure. S14. Cycling stability of BioMn600 electrode at 1 A g^{-1} for 1,000 cycles.

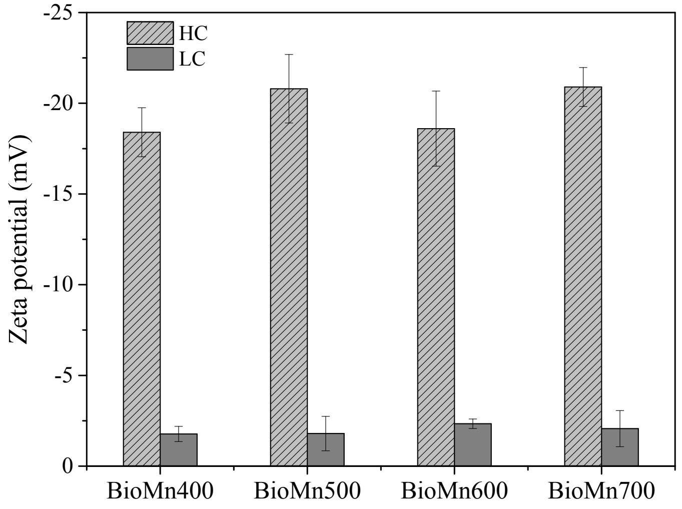

Figure. S15. Zeta potentials of BioMn400, BioMn500, BioMn600 and BioMn700 composites in LC (1 g L^{-1}) and HC (30 g L^{-1}) NaCl solutions.

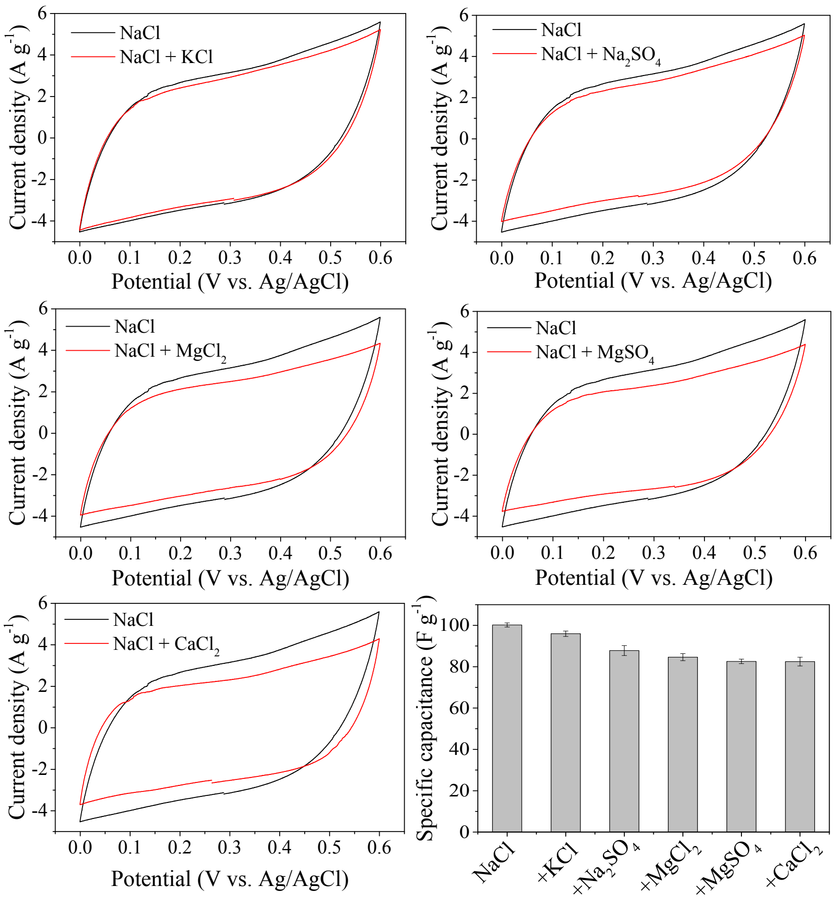

Figure. S16. Cyclic voltammograms and specific capacitances of BioMn600 electrode under pure NaCl solution, and the NaCl solution with another inorganic ion at scan rate of 5 mV s^{-1}.

Reference

1. Besha, A. T.; Tsehaye, M. T.; Aili, D.; Zhang, W.; Tufa, R. A., Design of Monovalent Ion Selective Membranes for Reducing the Impacts of Multivalent Ions in Reverse Electrodialysis. *Membranes* **2020,** *10* (1), 7.
2. Vermaas, D. A.; Veerman, J.; Saakes, M.; Nijmeijer, K., Influence of Multivalent Ions on Renewable Energy Generation in Reverse Electrodialysis. *Energ Environ Sci* **2014,** *7* (4), 1434-1445.
3. Kim, T.; Logan, B. E.; Gorski, C. A., High Power Densities Created From Salinity Differences by Combining Electrode and Donnan Potentials in a Concentration Flow Cell. *Energ Environ Sci* **2017,** *10* (4), 1003-1012.
4. Fortunato, J.; Peña, J.; Benkaddour, S.; Zhang, H.; Huang, J.; Zhu, M.; Logan, B. E.; Gorski, C. A., Surveying Manganese Oxides as Electrode Materials for Harnessing Salinity Gradient Energy. *Environ Sci Technol* **2020,** *54* (9), 5746-5754.
5. Post, J. W.; Hamelers, H. V.; Buisman, C. J., Energy Recovery From Controlled Mixing Salt and Fresh Water with a Reverse Electrodialysis System. *Environ Sci Technol* **2008,** *42* (15), 5785-5790.
6. Augustyn, V.; Simon, P.; Dunn, B., Pseudocapacitive Oxide Materials for High-rate Electrochemical Energy Storage. *Energ Environ Sci* **2014,** *7* (5), 1597-1614.
7. Wu, Y.; Jiang, G.; Liu, G.; Lui, G.; Cano, Z. P.; Li, Q.; Zhang, Z.; Yu, A.; Zhang, Z.; Chen, Z., A 3D Ordered Hierarchically Porous Non-carbon Electrode for Highly Effective and Efficient Capacitive Deionization. *J. Mater. Chem. A* **2019,** *7* (26), 15633-15639.
8. Zhou, Z.; Liu, T.; Khan, A. U.; Liu, G., Block Copolymer–based Porous Carbon Fibers. *Sci Adv* **2019,** *5* (2), eaau6852.
9. Liu, F.; Schaetzle, O.; Sales, B. B.; Saakes, M.; Buisman, C. J. N.; Hamelers, H. V. M., Effect of Additional Charging and Current Density on the Performance of Capacitive Energy Extraction Based on Donnan Potential. *Energ Environ Sci* **2012,** *5* (9), 8642-8650.
10. La Mantia, F.; Pasta, M.; Deshazer, H. D.; Logan, B. E.; Cui, Y., Batteries for Efficient Energy Extraction From a Water Salinity Difference. *Nano Lett* **2011,** *11* (4), 1810-1813.
11. Lee, J.; Yoon, H.; Lee, J.; Kim, T.; Yoon, J., Extraction of Salinity-Gradient Energy by a Hybrid Capacitive-Mixing System. *ChemSusChem* **2017,** *10* (7), 1600-1606.
12. Vermaas, D. A.; Bajracharya, S.; Sales, B. B.; Saakes, M.; Hamelers, B.; Nijmeijer, K., Clean Energy Generation Using Capacitive Electrodes in Reverse Electrodialysis. *Energ Environ Sci* **2013,** *6* (2), 643-651.
13. Sales, B. B.; Saakes, M.; Post, J. W.; Buisman, C. J. N.; Biesheuvel, P. M.; Hamelers, H. V. M., Direct Power Production From a Water Salinity Difference in a Membrane-modified Supercapacitor Flow Cell. *Environ Sci Technol* **2010,** *44* (14), 5661-5665.
14. Tan, G.; Zhu, X., Polyelectrolyte-Coated Copper Hexacyanoferrate and Bismuth Oxychloride Electrodes for Efficient Salinity Gradient Energy Recovery in Capacitive Mixing. *Energy Technology* **2020,** *8* (1), 1900863.
15. Smolinska-Kempisty, K.; Siekierka, A.; Bryjak, M., Interpolymer Ion Exchange Membranes for CapMix Process. *Desalination* **2020,** *482*, 114384.
16. Kim, T.; Rahimi, M.; Logan, B. E.; Gorski, C. A., Harvesting Energy From Salinity Differences Using Battery Electrodes in a Concentration Flow Cell. *Environ Sci Technol* **2016,** *50* (17), 9791-9797.
17. Tan, G.; Li, H.; Zhu, H.; Lu, S.; Fan, J.; Li, G.; Zhu, X., Concentration Flow Cells Based on Chloride-Ion Extraction and Insertion with Metal Chloride Electrodes for Efficient Salinity Gradient Energy Harvest. *ACS Sustain Chem Eng* **2018,** *6* (11), 15212-15218.
18. Zhu, H.; Xu, W.; Tan, G.; Whiddon, E.; Wang, Y.; Arges, C. G.; Zhu, X., Carbonized Peat Moss Electrodes for Efficient Salinity Gradient Energy Recovery in a Capacitive Concentration Flow Cell. *Electrochim Acta* **2019,** *294*, 240-248.
19. Tan, G.; Lu, S.; Fan, J.; Li, G.; Zhu, X., Chloride-ion Concentration Flow Cells for Efficient Salinity Gradient Energy Recovery with Bismuth Oxychloride Electrodes. *Electrochim Acta* **2019,** *322*, 134724.

20. Zhu, H.; Lai, J.; Arges, C. G.; Wang, Y.; Zhu, X., Engineering the Interlayer Spacing of Molybdenum Disulfide for Efficient Salinity Gradient Energy Recovery in Concentration Flow Cells. *Electrochim Acta* **2020**, 136103.
21. Tansel, B.; Sager, J.; Rector, T.; Garland, J.; Strayer, R. F.; Levine, L.; Roberts, M.; Hummerick, M.; Bauer, J., Significance of Hydrated Radius and Hydration Shells on Ionic Permeability During Nanofiltration in Dead End and Cross Flow Modes. *Sep Purif Technol* **2006,** *51* (1), 40-47.
22. Oh, Y.; Jeong, Y.; Han, S.-J.; Kim, C.-S.; Kim, H.; Han, J.-H.; Hwang, K.-S.; Jeong, N.; Park, J.-S.; Chae, S., Effects of Divalent Cations on Electrical Membrane Resistance in Reverse Electrodialysis for Salinity Power Generation. *Ind Eng Chem Res* **2018,** *57* (46), 15803-15810.
23. Pintossi, D.; Chen, C.-L.; Saakes, M.; Nijmeijer, K.; Borneman, Z., Influence of Sulfate on Anion Exchange Membranes in Reverse Electrodialysis. *npj Clean Water* **2020,** *3* (1), 1-10.
24. Lide, D. R., *CRC Handbook of Chemistry and Physics*. CRC press: **2004**; Vol. 85.
